# Frequency-Chirped Magic Angle Spinning Dynamic Nuclear Polarization Combined with Electron Decoupling

**DOI:** 10.1021/acs.jpclett.4c01075

**Published:** 2024-07-08

**Authors:** Marthe Millen, Nicholas Alaniva, Edward P. Saliba, Sarah A. Overall, Alexander Däpp, Ioannis Gr. Pagonakis, Snorri Th. Sigurdsson, Snædís Björgvinsdóttir, Alexander B. Barnes

**Affiliations:** † Institute of Molecular Physical Science, ETH Zurich, Vladimir-Prelog-Weg 2, 8093 Zurich, Switzerland; ‡ Faculty of Physical Sciences, University of Iceland, 107 Reykjavik, Iceland

## Abstract

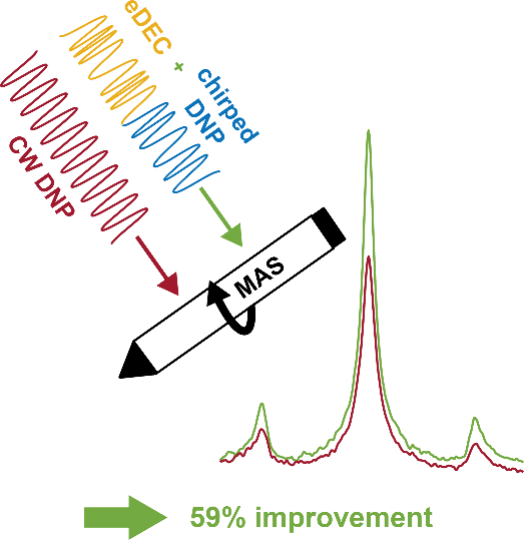

Magic angle spinning (MAS) dynamic nuclear polarization (DNP) increases the signal intensity of solid-state nuclear magnetic resonance. DNP typically uses continuous wave (CW) microwave irradiation close to the resonance frequency of unpaired electron spins. In this study, we demonstrate that frequency-chirped microwaves improve DNP performance under MAS. By modulating the gyrotron anode potential, we generate a train of microwave chirps with a maximum bandwidth of 310 MHz and a maximum incident power on the spinning sample of 18 W. We characterize the efficiency of chirped DNP using the following polarizing agents: TEMTriPol-1, AsymPolPOK, AMUPol, and Finland trityl. The effects of different chirp widths and periods are analyzed at different MAS frequencies and microwave powers. Furthermore, we show that chirped DNP can be combined with electron decoupling to improve signal intensity by 59%, compared to CW DNP without electron decoupling, using Finland trityl as a polarizing agent.

Magic angle spinning (MAS) solid-state nuclear magnetic resonance (NMR) is a powerful, nondestructive spectroscopic technique that can determine structural detail of solids with sub-Ångström precision. However, NMR is challenged by inherently low thermal spin polarization and a concomitant low sensitivity. Dynamic nuclear polarization (DNP) is a method that can substantially enhance NMR signal intensity by polarization transfer from highly polarized unpaired electron spins to nuclear spins. Typically, DNP is implemented using continuous, monochromatic microwave irradiation, also known as continuous wave (CW).^1−3^ In order to realize the full potential of DNP NMR, signals must be efficiently enhanced at ever higher magnetic fields, MAS frequencies, and sample temperatures.^4−8^ The enhancements of the most commonly used polarizing agents at moderate magnetic fields scale unfavorably with increasing magnetic fields.^9^ Two of the strategies that can be employed to improve DNP performance are the design of polarizing agents^8,10,11^ and the development of high frequency, high power, and preferably frequency-agile microwave sources^12−14^ for chirped/pulsed DNP. Pulsed DNP methods provide a promising approach to improve sensitivity at high magnetic fields.^15−18^ However, pulsed DNP at high magnetic field requires very high power microwaves and short pulses, not achievable with present technology. Compared to pulsed DNP methods that impose such strict requirements on the microwave source, chirped DNP can utilize more accessible broadband frequency-modulated microwave sources to increase DNP efficiency over CW DNP at high magnetic fields.^14,19−25^ Most microwave frequency-modulated DNP experiments have been performed under static conditions,^14,20−27^ with a few examples demonstrating chirped DNP under MAS.^28−30^

The description of the static DNP mechanisms such as solid effect (SE),^31,32^ cross effect (CE),^33−35^ and the Overhauser effect^36^ differs from the description under MAS. The energy spin states of the electron−electron nuclear three spin system of the CE are modulated by sample rotation due to the anisotropic nature of the g-tensor, dipolar, and hyperfine interactions.^37−40^ As a result, the interactions between the spins in the three spin system become separated in time under MAS. This leads to avoided level crossings (also called rotor events)^38^ that allow population exchange if the transitions between states are adiabatic. The efficiency of the polarization transfer by the CE mechanism is governed by three rotor events which are electron microwave encounters, electron–electron dipolar anti-crossings and CE anti-crossings.^37,38^ The probability of the rotor events following an adiabatic pathway can be described by the Landau–Zener equation,^41^ given by
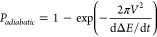
1where *V* corresponds to the mixing interaction connecting the two populations and *dΔE*/*dt* to the energy crossing rate. Depending on the rate of change of the energy states *dΔE*/*dt* and the magnitude of the perturbation *V*, the transition can be adiabatic, diabatic, or something in between. For illustration purposes, the simulated evolution of the electron frequencies during one rotor period is shown in Figure 1a. In addition to the time dependence of the rotor events under MAS, frequency modulation of the microwaves leads to another time dependence of the electron microwave encounters. Microwave frequency chirps increase the number of electron microwave encounters and thus provide a strategy to improve the efficiency of CW DNP.

**Figure 1:**
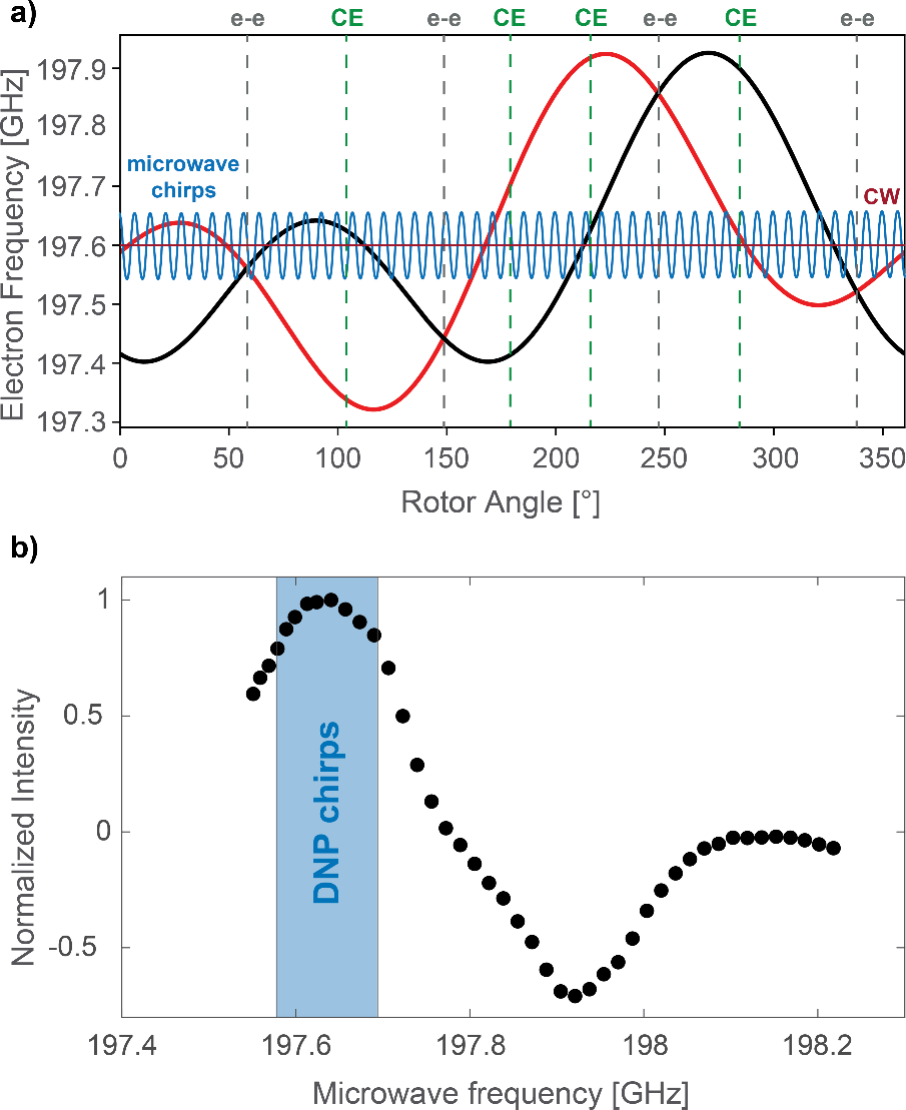
(a) Simulation of the electron frequencies (black and red curves) of one crystal orientation of a nitroxide biradical during one rotor period at a field of 7 T. The electron–electron dipolar anti-crossings (e-e) and CE anti-crossings (CE) are indicated with vertical lines. The horizontal red line represents CW irradiation, while sinusoidal microwave chirps with a bandwidth of 114 MHz and period of 20 μs are shown at 1 kHz MAS (see SI for further information). (b) ^1^H DNP enhancement profile of the TEMTriPol-1 sample under MAS (1 kHz). The highlighted section corresponds to the frequency range (114 MHz) covered by DNP chirps that were centered around the positive DNP condition at 197.636 GHz.

Here, we investigate the dependency of frequency-chirped DNP across commonly used polarizing agents and various MAS frequencies. Furthermore, the relative importance of the different sweep parameters, such as the sweep width, the sweep period, and the microwave power on the resulting DNP signal is characterized. We employ sinusoidal frequency modulations of the microwaves, defined by a bandwidth in MHz and a period in μs. To generate the chirps, a high voltage amplifier (HVA) that uses the input from an arbitrary waveform generator (AWG) is connected to the gyrotron anode. This combination makes it possible to modulate the voltage of the gyrotron anode in the kV range, which results in a frequency-modulated microwave output with a specific bandwidth in the MHz range.^28^

The following radicals were chosen for the analysis of chirped DNP under MAS: the broad line bis-nitroxides AMUPol^42^ (10 mM) and AsymPolPOK^43^ (10 mM), the heterobiradical TEMTriPol-1^44,45^ (12 mM), and the narrow line Finland trityl (40 mM). A comparison between the concentrations of 10 mM and 20 mM for AsymPolPOK and AMUPol can be found in Figure S6 in the SI. The radicals were added to ^13^C,^15^N-labeled urea in a glass-forming glycerol/water matrix. All DNP experiments were conducted at a field of 7 T, at sample temperatures of approximately 100 K. More information on the sample preparation and DNP experiments can be found in the Supporting Information (SI).

We focus first on the CE trityl-nitroxide biradical TEMTriPol-1 as improved enhancements using frequency-modulated DNP under MAS for TEMTriPol-1 have been observed previously at spinning frequencies of 3 and 4.5 kHz.^28−30^Figure 2a shows how chirped DNP leads to an improved signal intensity of up to 119% compared to CW DNP across six different MAS frequencies, ranging from 500 Hz to 8 kHz. The percentages shown above the bars in Figure 2 represent the relative signal increase with chirped DNP compared to CW DNP at each MAS frequency. By means of a GHz detection system (more details provided in the SI) the microwave frequency can be recorded during the experiments, which allows precise monitoring of the generated chirps. For the TEMTriPol-1 sample, input sweep parameters of 1 V and 50 kHz (amplitude and frequency of sine wave) were used and a microwave bandwidth of 114 MHz with a period of 20 μs was measured using the GHz detection circuit. The bandwidth covered by these microwave sweeps, with a microwave power of 9 W incident on the sample, is highlighted in blue on the DNP enhancement profile of the TEMTriPol-1 sample depicted in Figure 1b. The corresponding bandwidths in MHz of all chirps are reported in Table S4 and Figure S3 in the SI. The center frequencies of the chirps are indicated in the caption of Figure 2 and were carefully chosen based on the DNP profile for each radical.

**Figure 2:**
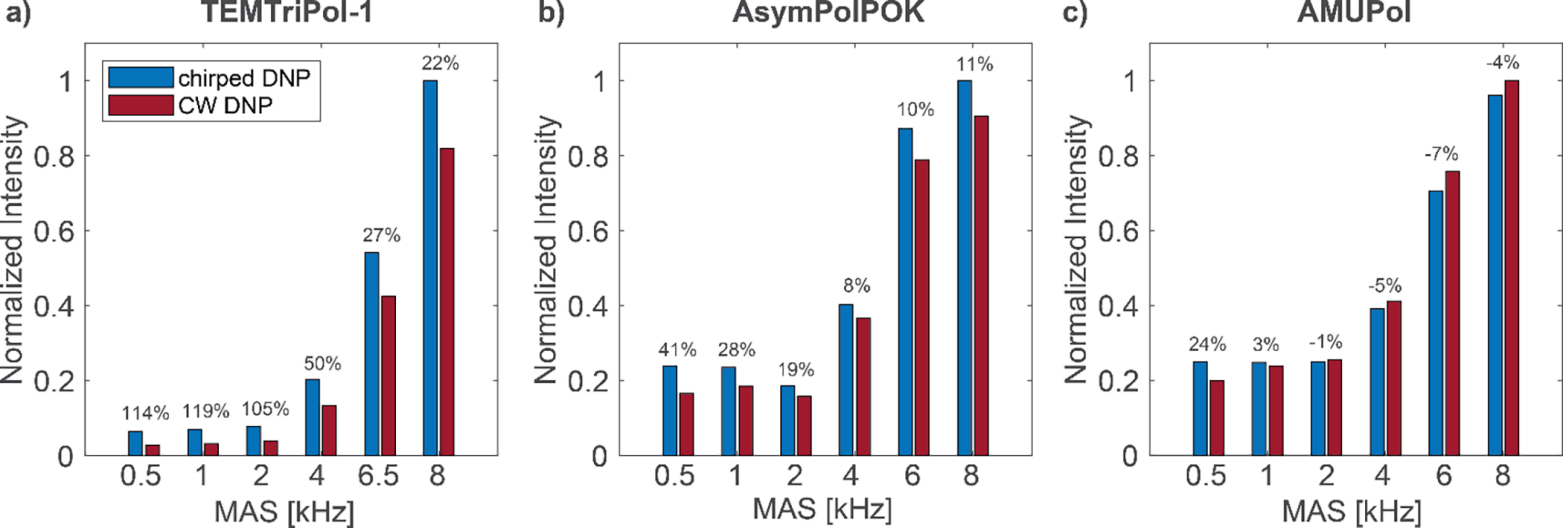
Normalized DNP enhanced ^1^H signal intensities of the (a) TEMTriPol-1, (b) AsymPolPOK, and (c) AMUPol samples at different MAS frequencies without (CW DNP) and with sinusoidal microwave chirps (chirped DNP). The microwave sweeps were centered around 197.636, 197.577, and 197.732 GHz for the TEMTriPol-1, the AsymPolPOK, and the AMUPol samples, respectively. The chirped ^1^H DNP enhancements at 8 kHz MAS were equal to 52 for the TEMTriPol-1 sample, 27 for the AsymPolPOK, and 55 for AMUPol (see Tables S1, S2, and S3 in SI for all enhancements). The relative signal increase using chirped DNP compared to CW DNP is indicated in percentages above the bars (see also Figure S5 in SI).

TEMTriPol-1 exhibits a narrow and a broad linewidth component in its electron paramagnetic resonance (EPR) spectrum, originating from the trityl and nitroxide moieties of the radical, respectively.^44^ This enables the selective saturation of one of the two electrons and conserves a large polarization differential between them, which is critical for an efficient CE DNP performance. Chirped DNP allows broadband electron spin saturation compared to CW irradiation. Both the number of rotor events per unit time (increasing with faster MAS) and the adiabatic probability *P*_*adiabatic*_ of the events affect the CW DNP enhancement under MAS.^37,38,46^ As can be inferred from eq 1, higher MAS frequency results in a higher energy crossing rate which leads to a lower probability of adiabaticity. From 0.5 kHz to 8 kHz MAS we observe an expected increase in CW DNP enhancement which suggests that at these spinning frequencies the *P*_*adiabatic*_ is still high enough to benefit from the higher number of rotor events per unit time. When the chirp parameters are kept constant, more DNP chirps take place in one rotor period at lower spinning frequencies, which leads to more electron microwave encounters (see Figure S4b in SI). The number of chirps per rotor period, τ_*rotor*_/τ_*chirp*_, where τ_*rotor*_ is the rotor period and τ_*chirp*_ is the chirp period (here 50 kHz), ranges from 100 chirps at 500 Hz MAS, to 6.3 chirps at 8 kHz MAS frequency. This can be seen in Figure 2a, where the percentage signal improvement of chirped DNP over CW DNP decreases when going to faster sample spinning (from 114% increase at 500 Hz MAS to 22% at 8 kHz MAS). At higher MAS frequencies the improvement of chirped DNP over CW DNP is less pronounced as the self-chirping effect of the faster sample spinning already leads to more electron microwave encounters per unit time.

Next, we consider the effect of microwave frequency chirps on two different CE nitroxide biradicals, AsymPolPOK (Figure 2b) and AMUPol (Figure 2c). The AsymPolPOK sample shows improvements in enhancement at all rotor spinning frequencies when frequency chirps are applied. For the AsymPolPOK sample, 12 W chirps over 137 MHz with a period of 10 μs (4 V, 100 kHz input parameters) were chosen. The dependence of the microwave power on chirped and CW DNP was analyzed at two different MAS frequencies (see Figure S7c,d in SI) and it was observed that higher power results in a larger difference between the chirped and CW DNP signal.

The improvements in enhancement for the AsymPolPOK sample follow the same trend as for the TEMTriPol-1 sample, but are not as pronounced. This could be attributed to the broader EPR linewidth of AsymPolPOK, as the absence of a narrow component makes it more challenging to induce additional enhancements using microwave chirps. As has been observed for the TEMTriPol-1 sample, the percentage in signal improvement decreases at higher MAS frequencies for AsymPolPOK.

Under the experimental conditions used here, microwave chirps only provided improvements in DNP enhancements of the AMUPol sample when the MAS frequency was low (Figure 2c). For the AMUPol sample the following chirp parameters were used: a bandwidth of 80 MHz, a chirp period of 10 μs (2 V, 100 kHz input) and 9 W incident power on the sample. Although they are both bis-nitroxides, AMUPol and AsymPolPOK are not necessarily expected to exhibit the same behavior since they differ significantly in their design parameters.^47,48^ These include different relaxation properties,^47,49^ different g-tensor anisotropies, and solvent accessibilities.^50,51^ For example, AsymPolPOK has a shorter electron spin–lattice relaxation time, T_1e_ than AMUPol.^47^ Since frequency-chirped irradiation can allow electron microwave encounters to occur more frequently in one rotor period this can reduce the detrimental effect of the electron spin relaxation back to thermal equilibrium under CW MAS DNP.^30^ AMUPol exhibits nuclear depolarization in the absence of microwaves^39,52,53^ whereas depolarization effects have been limited for AsymPolPOK and TEMTriPol-1 by design.^43,54^ AMUPol also has an even broader EPR linewidth than AsymPolPOK^43,49^ with the two nitroxide electrons manifesting similar EPR linewidths, therefore it is challenging to selectively saturate one of the two electron spins using microwave sweeps.^55^ However, saturation of one of the electrons is crucial as it leads to a larger polarization difference between the two electrons which drives the CE anti-crossings transferring polarization to the nucleus.

Depending on the strength of the electron–electron dipolar coupling, exchange interaction, MAS frequency, and molecular orientation, the probability of the electron–electron dipolar anti-crossings being nonadiabatic might be high enough to result in only partial polarization exchange. The balance between the electron–electron dipolar coupling and exchange interaction in AMUPol and AsymPolPOK is different, and the smaller electron–electron dipolar coupling of AMUPol can lead to nonadiabatic electron–electron dipolar anti-crossings.^56^ This leads to an absence of a polarization differential between the two electrons spins, which gives rise to nuclear depolarization if the Boltzmann thermal polarization difference between the two electrons is less than the thermal polarization of the coupled nucleus. Indeed, independent of microwave irradiation, loss of nuclear polarization to the coupled electrons occurs in the absence of this electron polarization differential if the matching condition is fulfilled.

A possible explanation for the differences observed between the AsymPolPOK and AMUPol samples could be that in the case of AMUPol, when electron–electron dipolar anti-crossings are nonadiabatic, microwave chirps lead to more electron microwave encounters on both electrons which combined with only partial electron–electron exchange leads to enhanced reduction of the electron polarization differential. In this case CW irradiation, which causes less electron microwave encounters than chirps, is beneficial as a larger polarization difference can be maintained compared to the chirps. This becomes even more pronounced at higher MAS frequencies as the adiabatic probability *P*_*adiabatic*_ of the rotor events decreases. In order to verify this hypothesis, further experiments would need to be performed, preferably complemented by simulations.

Chirped DNP experiments also improve enhancements on a Finland trityl sample. For the narrow-line Finland trityl sample at the relatively high concentration of 40 mM, both the CE and SE DNP mechanisms are present (Figure S10 in SI).^57,58^ A 16% increase in ^13^C signal intensity using chirps compared to CW irradiation was observed at a MAS frequency of 4 kHz.

In order to optimize the enhancements obtained with chirped DNP, the incident microwave power, the center microwave frequency, the sweep bandwidth, and sweep period are varied. The optimal chirp parameters not only depend on the radical and its relaxation properties but also on the MAS frequency. To choose appropriate chirp parameters for a specific radical at a certain MAS frequency, DNP experiments with sweep amplitudes ranging from 0.1 to 3 V (bandwidths 10–310 MHz) as well as sine frequencies between 5 kHz and 150 kHz (periods 200–6.6 μs) were compared. Examples of such optimizations are shown in Figure 3, where the normalized DNP signal intensities are presented for the TEMTriPol-1 sample spinning at 8 kHz (Figure 3a,b) and for the Finland trityl sample at a MAS frequency of 4 kHz (Figure 3c). The intensities in Figure 3 are compared to the DNP signal intensity without microwave chirps, hence values higher than 1 indicate that the frequency-chirped DNP experiments result in a signal improvement over CW DNP. While Figure 3a,c provide an overview of the optimal input sweep parameters, Figure 3b shows the relative performances of different chirp bandwidths at different chirp periods (also Figure S9 in SI for the Finland trityl sample). For all radicals, more than one set of parameters exists for which a signal boost over CW DNP is observed. For the TEMTriPol-1 sample (Figure 3a) all parameter sets result in an improvement in intensity except for the slow sweeps of 5 kHz (200 μs period). Moreover, a sweep bandwidth of around 100 MHz provides the best improvements over CW DNP. This approximately corresponds to the EPR linewidth of the trityl electron spins of TEMTriPol-1.^28,44^ On the other hand, for the Finland trityl sample (Figure 3c) large bandwidths (high amplitudes at longer periods) do not lead to an improvement in DNP signal and even show very little signal enhancement compared to the experiment without microwaves. This could be attributed to the relatively narrow DNP profile of the Finland trityl radical (see Figure S10 in SI), indicating that a large-amplitude chirp covers a frequency range that is broader than the linewidth, thus the microwaves spend more time off resonance, which in turn lowers the obtained enhancement. Moreover, the chirps could lead to saturation of not only the forbidden double quantum, but also allowed single quantum transition, which also results in a lower DNP enhancement. Additional sweep parameter optimizations for the TEMTriPol-1, AsymPolPOK and AMUPol samples at various MAS frequencies are shown in Figure S8 in the SI.

**Figure 3:**
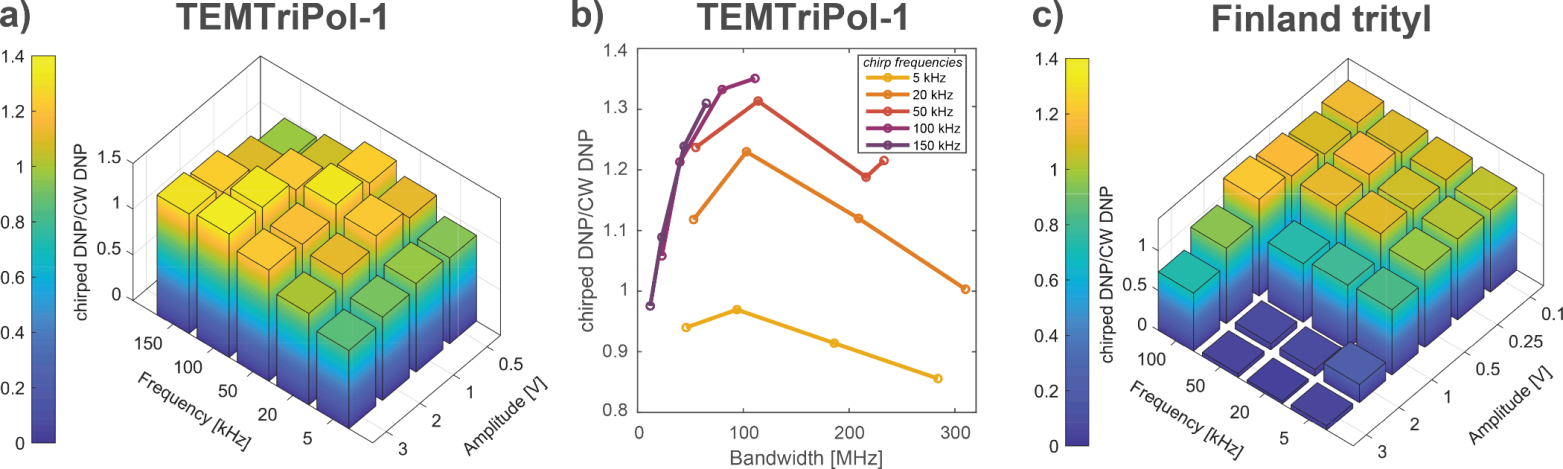
Optimization of signal intensities of the frequency-chirped DNP experiments compared to CW DNP using different sweep parameters for the (a) and (b) ^1^H signal intensities of the TEMTriPol-1 sample at 8 kHz MAS frequency and (c) ^13^C signal intensities of the Finland trityl sample spinning at 4 kHz. Illustrations (a) and (c) are plotted using the chirp input parameters (amplitude and frequency), while (b) shows the same result as in (a) but as a function of the bandwidth covered by the microwave chirps and observed experimentally.

It should be noted that the HVA limits the amplitudes set by the AWG at high frequency sine modulations (100 kHz and 150 kHz) as can be seen in Figure 3b and Figure S9 in SI. As a result, microwave frequency sweeps with the correct period but with a smaller bandwidth are obtained (see also Table S4 in SI). Nevertheless, we show which sweep parameters result in the highest signal improvement within the technical limitations of the HVA. The obtained bandwidths were closely monitored by an oscilloscope and the GHz detection system. In this work only sinusoidally shaped modulations were used in order to restrict the wide parameter space, but more sophisticated modulations can in principle be implemented without making changes to the experimental setup.

Using an in-house designed semiconductor switching unit that enables controlled alternation between two AWG channels (see SI for more details), DNP chirps can be combined with electron decoupling as shown schematically in the pulse sequence in Figure 4a. Electron decoupling (or hyperfine decoupling)^12,59−63^ is employed during signal acquisition and consists of sweeping the microwaves over the electron resonance, which can lead to improvements in signal intensity. This method is similar to ^1^H heteronuclear decoupling in NMR experiments. The DNP pulse sequence in Figure 4a begins with a saturation train to ensure zero residual magnetization at the start of the experiment. On the electron channel, electron decoupling chirps were applied during the acquisition time while DNP chirps were repeated for the entire polarization delay, which was 3 s for the Finland trityl sample. Both the DNP chirps and electron decoupling consisted of sinusoidally frequency-swept microwaves. The DNP chirps were centered at the positive DNP condition of the sample, while the electron decoupling was centered at the EPR resonance frequency of Finland trityl. The input parameters for electron decoupling can be optimized in the same manner as the DNP chirps (Figure S8f in the SI). A maximum improvement in intensity with electron decoupling was observed for a modulation with a bandwidth of 111 MHz and a period of 10 μs (3 V, 100 kHz).

**Figure 4:**
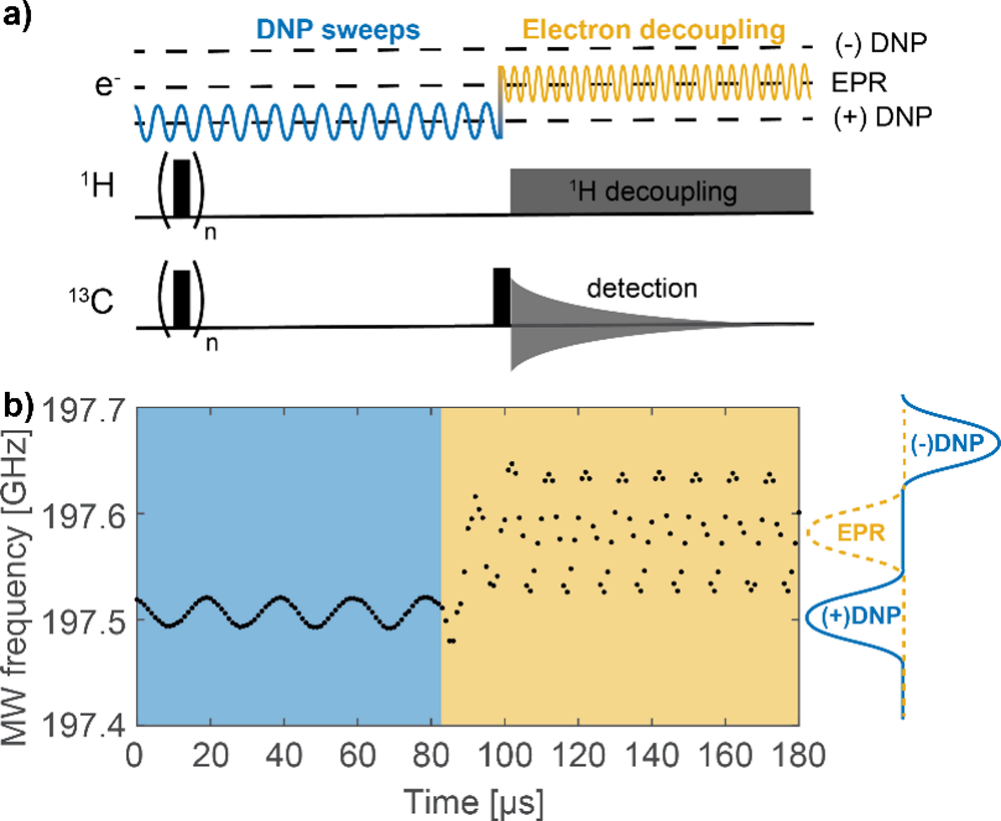
(a) Pulse sequence used for frequency-chirped DNP experiments combined with electron decoupling. (b) Microwave frequency recorded with the GHz detection circuit during the transition section between the DNP chirps and electron decoupling of the pulse sequence shown in a. For the DNP sweeps, the gyrotron anode voltage was modulated with a 50 kHz, 0.25 V sine wave (20 μs period, 30 MHz bandwidth), while a 100 kHz, 3 V sine was used for the electron decoupling (10 μs period, 111 MHz bandwidth). The DNP sweeps were centered around 197.509 GHz, and the electron decoupling was centered at the electron resonance frequency of Finland trityl, 197.582 GHz.

Figure 4b shows the microwave frequency modulations during the experiment recorded with the GHz detection system. The microwaves exactly follow the gyrotron anode voltage modulation that is controlled by the AWG settings. The bandwidth covered by the 0.25 V, 50 kHz DNP sweeps was 30 MHz with a period of 20 μs (blue shaded area in Figure 4b) while for electron decoupling, a range of 111 MHz was covered and a period of 10 μs was recorded (3 V, 100 kHz) (yellow shaded area in Figure 4b). As mentioned above, due to instrumentation limitations the voltage of 3 kV could not be reached with a 100 kHz sine, and as a result voltage amplitude reaching the gyrotron anode was lower than 3 kV, leading to a lower bandwidth than for sinusoidal sweeps of the same amplitude with a longer period.

The combination of DNP sweeps and electron decoupling leads to both effects approximately adding up, as demonstrated in Figure 5a. For the Finland trityl sample spinning at 4 kHz, the DNP chirps led to an improvement in signal intensity of 16%, and the electron decoupling to a signal boost of 39%. Combining the two by using the pulse sequence in Figure 4a, a signal enhancement of 59% over the CW, nonelectron-decoupled experiment was obtained.

**Figure 5:**
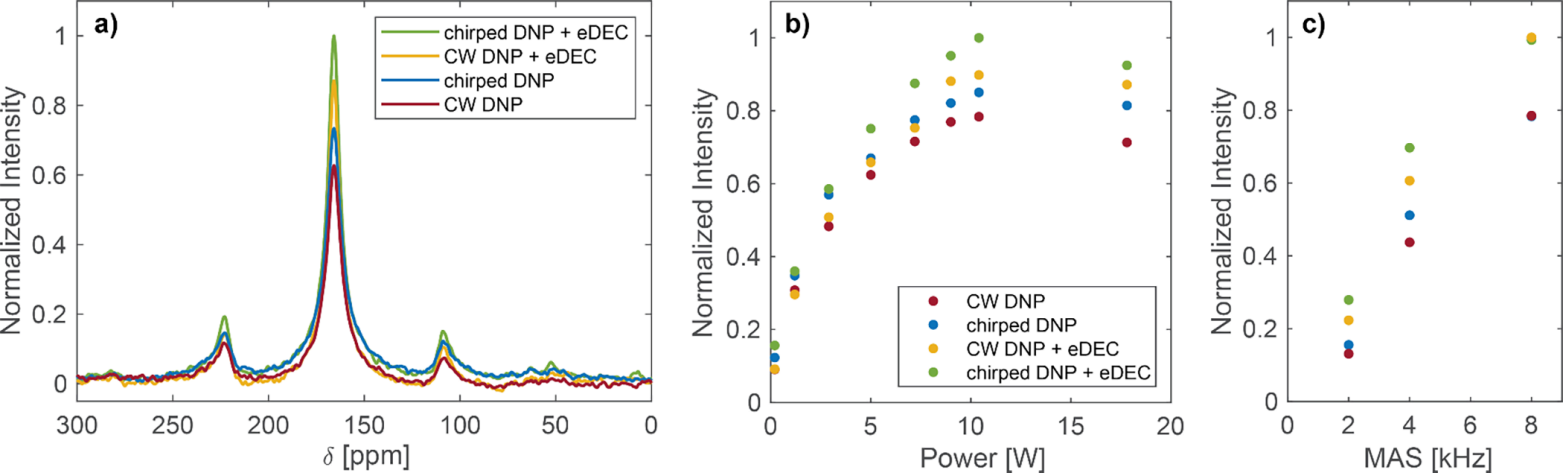
(a) Normalized ^13^C urea signal of the Finland trityl sample for: CW DNP (red), chirped DNP using 20 μs sweeps with a bandwidth of 30 MHz (blue), CW DNP with electron decoupling (eDEC) using a 111 MHz broad, 10 μs modulation (yellow) and DNP chirps combined with electron decoupling (green). (b) Dependence of the normalized ^13^C signal intensity of urea doped with Finland trityl on different microwave powers incident on the sample. (c) Normalized ^13^C signal intensities of the same sample as a function of the MAS frequency. At 8 kHz MAS the yellow and green points overlap, as well as the red and blue points. The same color code is used in (a), (b), and (c).

We note that for all DNP experiments, circularly polarized microwaves generated with a Martin-Puplett interferometer were used, as an improved enhancement of 34%^64^ was obtained with circular compared to linear polarization.^65^ The quasi optical system, including the interferometer, can also be used to attenuate the microwave power generated by the gyrotron (see SI). Figure 5b shows the microwave power dependence of the different experiments. The sweep parameters were kept constant, and are the same as described above and recorded in Figure 4b. It can be concluded that up until CE saturation, higher microwave power generally leads to higher enhancement for both CW DNP and chirped DNP. However, electron decoupling requires a certain amount of microwave power to improve the signal, while the DNP chirps result in a small signal improvement even at lower power, as can be seen at 1 W in Figure 5b.

In addition, the effect of the DNP sweeps combined with electron decoupling was analyzed at different MAS frequencies (Figure 5c). The improvement in signal intensity was clearly observed at 2 kHz and at 4 kHz MAS frequency, however at 8 kHz no improvement was observed with DNP chirps (improvement was still observed with electron decoupling). This could be because the sweep parameters were not sufficiently optimized at 8 kHz.

In conclusion, this study shows how controlled manipulation of electron spin polarization, achieved by microwave frequency chirps, can lead to improved enhancements compared to CW DNP. Optimized frequency-modulated microwaves can boost the signal intensity of DNP under MAS through sinusoidal frequency sweeps around the positive DNP enhancement during the signal build-up time (chirped DNP), and around the EPR resonance for electron decoupling during the acquisition time. Combined frequency-swept DNP and electron decoupling leads to an increase in signal of 59% for the Finland trityl sample. TEMTriPol-1 and AsymPolPOK show improved enhancements with chirped DNP from low spinning frequencies up to 8 kHz MAS. For AMUPol, chirped DNP results in an increased signal compared to CW DNP for the first time under MAS, however only at MAS frequencies lower than 2 kHz.

Chirped DNP enhancements under MAS are a complex interplay between several factors such as the incident microwave power, the center microwave frequency, the microwave sweep width and period, the radical and its properties, and the MAS frequency. However, we do find that more than one combination of chirp amplitude and frequency can lead to an improved enhancement. With the development of higher power microwave sources with faster frequency tunability, frequency-modulated DNP under MAS is expected to improve further.

## Supporting Information Available

Sample preparation, DNP NMR experiment details, schematic of GHz detection circuit, frequency ranges covered by microwave chirps, performance of chirped DNP at different radical concentrations and microwave power, additional sweep parameter optimizations, parameters used for simulations, and details on semiconductor switch (PDF)
