# Supplementary material for: Frequency-Chirped Magic Angle Spinning Dynamic Nuclear Polarization Combined with Electron Decoupling
Source: J Phys Chem Lett. 2024 Jul 8;15(28):7228–35. doi: 10.1021/acs.jpclett.4c01075 (PMC11261599; doi:10.1021/acs.jpclett.4c01075)
Supplement: Supplementary file 1 — jz4c01075_si_002.pdf [file jz4c01075_si_002.pdf]

Supporting Information: Frequency-Chirped Magic Angle Spinning Dynamic Nuclear Polarization Combined with Electron Decoupling

Marthe Millen,† Nicholas Alaniva,† Edward P. Saliba,† Sarah A. Overall,† Alexander Däpp,† Ioannis Gr. Pagonakis,† Snorri Th. Sigurdsson,‡ Snædís Björgvinsdóttir,*,† and Alexander B. Barnes*,†

†*Institute of Molecular Physical Science, ETH Zurich, Vladimir-Prelog-Weg 2, 8093 Zurich, Switzerland*

‡*Faculty of Physical Sciences, University of Iceland 107 Reykjavik, Iceland*

E-mail: snaedis.bjoergvinsdottir@phys.chem.ethz.ch; alexander.barnes@phys.chem.ethz.ch

Sample preparation

The TEMTriPol-1 (12 mM) sample was prepared by adding the biradical to a 4 M solution of ^{13}C,^{15}N-labelled urea in DNP juice (glycerol:D_2O:H_2O, 60:30:10, v%). The AsymPolPOK and AMUPol samples were prepared to have a 10 mM concentration of the radical in DNP juice with a ^{13}C,^{15}N-labelled urea concentration of 4 M and 2 M, respectively. The Finland Trityl sample consists of 40 mM Finland trityl and 4 M ^{13}C,^{15}N-labelled urea in 50% DNP juice and 50% deuterated DNP juice (glycerol-d8:D_2O, 60:40, v%).

DNP NMR experiments

Solid-state NMR experiments were carried out on a Bruker Avance III spectrometer, using a 7 T Oxford magnet and a frequency-agile 198 GHz gyrotron. The samples were spun at around 100 K, using a custom-built cryogenic DNP setup including a 4-channel 3.2 mm MAS NMR probe.[1] Signal enhancement values were calculated as $\epsilon = I_{\mathrm{on}}/I_{\mathrm{off}}$ where I is the signal intensity. The ^1H enhancements for the different samples are given in Tab. S1, S2, and S3 where the columns represent the different MAS frequencies while the rows correspond to the enhancements with and without microwave chirps. The relative signal improvements indicated in % in Fig. 2 and Fig. S6 were calculated by $(I_{\mathrm{chirped\ DNP}}/I_{\mathrm{CW\ DNP}})/I_{\mathrm{CW\ DNP}} \cdot 100$. For the DNP experiments shown in Fig. 2, the microwave power at the sample was estimated to be about 9-12 W based on a power measurement above the 7 T magnet and a calculated loss over the last microwave transmission section.

Tab. S1: Measured ^{1}H enhancements for the TEMTriPol-1 sample.

	**0.5 kHz**	**1 kHz**	**2 kHz**	**4 kHz**	**6.5 kHz**	**8 kHz**
ϵ_{chirps}	18	18	20	34	47	52
ϵ	8	8	10	22	37	42

Tab. S2: ^{1}H enhancements obtained for the AsymPolPOK sample.

	0.5 kHz	1 kHz	2 kHz	4 kHz	6 kHz	8 kHz
ϵ_{chirps}	21	20	21	23	32	27
ϵ	14	16	18	21	29	24

Tab. S3: ^{1}H enhancements measured on the AMUPol sample.

	0.5 kHz	1 kHz	2 kHz	4 kHz	6 kHz	8 kHz
ϵ_{chirps}	35	34	36	52	65	53
ϵ	28	33	36	54	70	55

For the DNP experiments using biradicals, direct excitation of ^{1}H nuclei with a $\pi/2$-pulse was used to record NMR spectra. For the Finland trityl, direct excitation of ^{13}C nuclei with ^{1}H decoupling was used. For all experiments, saturation pulse trains on the respective channels were applied preceding the excitation pulse. Build-up times were measured with a saturation recovery sequence and fitted to an exponential function. The polarization delay was set to at least 1.3 times the build-up time for TEMTriPol-1, AMUPol, and AsymPolPOK. For the Finland trityl sample a polarization delay of 3 s was used but the build-up time is about ten times longer. The spectra were referenced to literature values of urea.

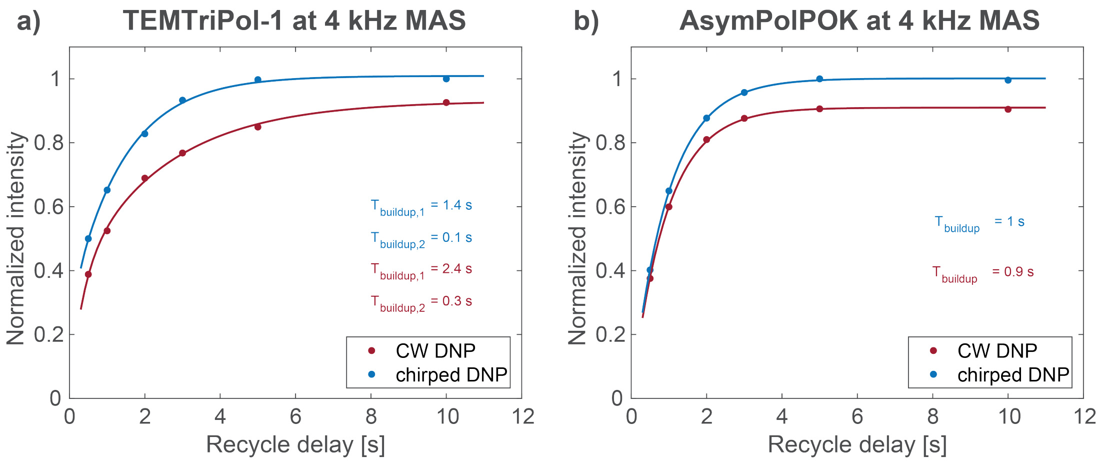

Fig. S1: Polarization build up curves with and without microwave chirps for **a)** the 12 mM TEMTriPol-1 and **b)** the 10 mM AsymPolPOK samples spinning at 4 kHz. The curves are fit either with a single exponential or with the sum of two exponentials.

GHz detection circuit

The GHz detection circuit consists of only a few components as shown in Fig. S2. The microwaves (around 198 GHz with a wavelength of around 1.51 mm) generated by the gyrotron are first down-converted by a subharmonic mixer that produces the sum and the difference of the microwave signal with a reference signal coming from a local oscillator. Then the high frequency signal (sum) created by the mixer is eliminated by a low pass filter. Before digitization by an analog-to-digital converter, the signal is amplified.

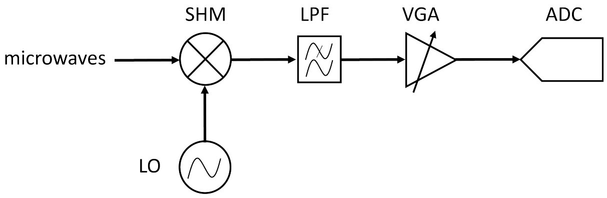

Fig. S2: Schematic illustration of the GHz detection system. SHM stands for subharmonic mixer, LO for local oscillator, LPF for low pass filter, VGA for variable gain amplifier, and ADC for analog-to-digital converter.

Frequency range covered by microwave chirps

Tab. S4: Predicted frequency range in MHz covered by the sinusoidal microwave chirps which are characterized by an AWG input amplitude in V (rows) and a input frequency in kHz (columns). The four values indicated in parentheses were recorded with the GHz detection circuit while all other values are predicted from the voltage reaching the gyrotron anode that was monitored by an oscilloscope.

	**5 kHz**	**20 kHz**	**50 kHz**	**100 kHz**	**150 kHz**
**0.1 V**	9 MHz	11 MHz	12 MHz	5 MHz	-
**0.25 V**	24 MHz	27 MHz	29 (30) MHz	11 MHz	-
**0.5 V**	46 MHz	53 MHz	55 MHz	23 MHz	12 MHz
**1 V**	94 MHz	103 MHz	116 (114) MHz	42 (40) MHz	23 MHz
**2 V**	186 MHz	209 MHz	216 MHz	80 MHz	44 MHz
**3 V**	284 MHz	310 MHz	233 MHz	118 (111) MHz	65 MHz
**4 V**	-	-	-	137 MHz	-

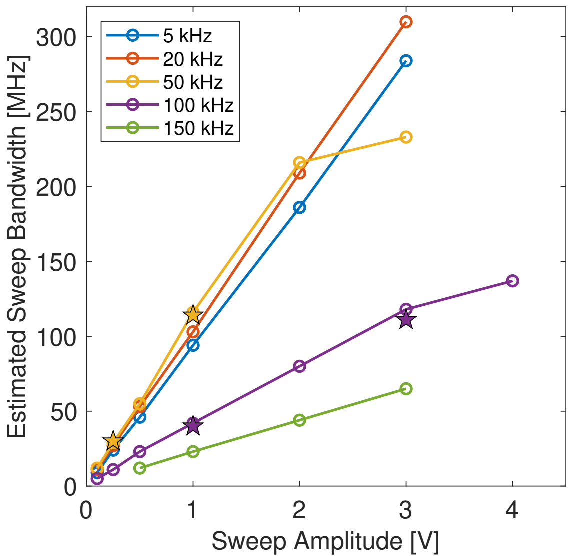

Fig. S3: Estimated frequency range covered by the frequency chirps based on AWG input parameters and the voltage on the gyrotron anode. The solid lines connect the dots. The stars represent the experimentally determined bandwidths using the GHz detection system (values in parentheses in Tab. S4). The high-voltage amplifier has a limited slew rate, hence the bandwidths become truncated if the chirp input parameters are high amplitudes and high frequencies.

Simulated electron frequencies under MAS

The g-tensor principal axis values used for the calculations in Fig. 1 a) and Fig. S4 a) were: $g_{xx} = 2.00925$, $g_{yy} = 2.00619$, $g_{zz} = 2.00212$ and Euler angles defining the relative orientation between two g-tensors: $\alpha = 58°$, $\beta = 57°$, $\gamma = 126°$.[2] For the trityl-nitroxide calculations in Fig. S4 b) the following parameters were used: g-tensor principal axis values of $g_{xx,1} = 2.0098$, $g_{yy,1} = 2.0064$, $g_{zz,1} = 2.0024$ and $g_{xx,2} = 2.0034$, $g_{yy,2} = 2.0031$, $g_{zz,2} = 2.0027$ for the nitroxide and trityl electron spins, respectively. The Euler angles were set to: $\alpha = 90°$, $\beta = 90°$, $\gamma = 90°$.[3]

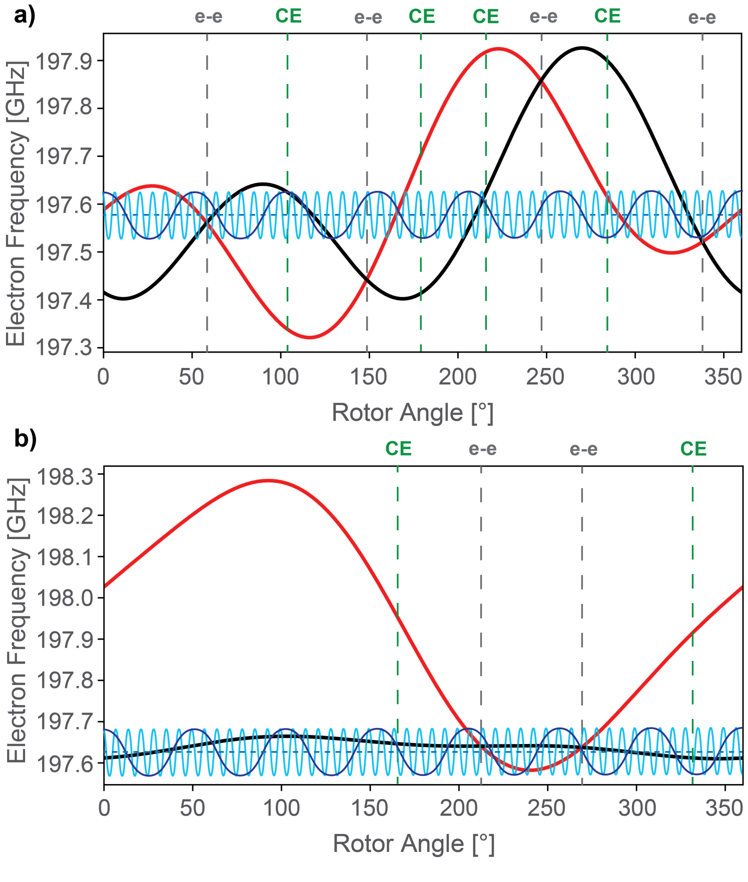

Fig. S4: Simulated oscillations of the electron frequencies (black and red curves) of **a)** a nitroxide biradical and **b)** a trityl-nitroxide biradical during one rotor period. When using the same sweep parameters (amplitude and frequency), the light blue sinusoidal modulation correspond to a MAS frequency of 1 kHz while dark blue corresponds to 8 kHz MAS.

Relative improvement of chirped DNP versus CW DNP

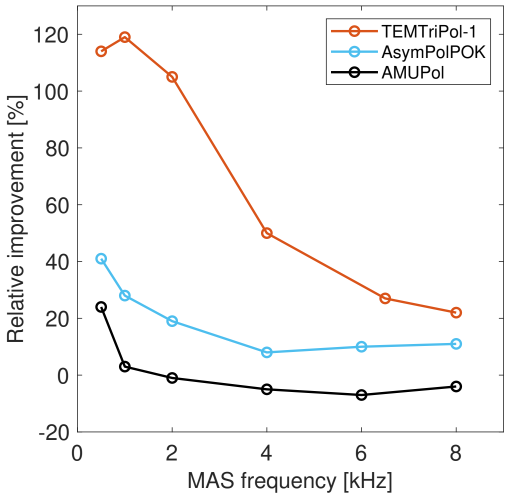

Fig. S5: Relative signal increase obtained by applying chirps compared to CW DNP for 12 mM TEMTriPol-1, 10 mM AsymPolPOK, and 10 mM AMUPol as a function of the spinning frequency. The lines are a guide for the eye. Percentages presented here are indicated above the bars in Fig. 2.

Semiconductor switch

The semiconductor switching and gating unit, used to switch between the two outputs on the AWG, consists of a four-channel integrated circuit switch MAX314 from Maxim and inverter gates SN74LVC1G04 from Texas Instruments controlling the switch. The switch is configured with two separate inputs as gate and as a changeover switch. The analog signal bandwidth is 66 MHz. Switching and gating is achieved at 50 ns rise and fall time.

Chirped DNP at different radical concentrations

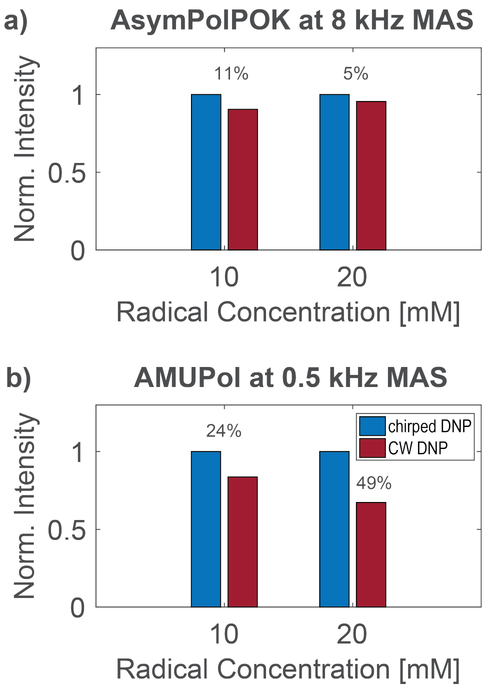

Fig. S6: Normalized DNP enhanced ^{1}H signal intensities of **a)** the AsymPolPOK samples spinning at 8 kHz, and **b)** the AMUPol samples spinning at 0.5 kHz using two different radical concentrations. CW DNP is compared to chirped DNP where sinusoidal microwave chirps are employed. Above the bars, the relative signal increase using chirped DNP compared to CW DNP is indicated in percentages. A bandwidth of 137 MHz and chirp period of 10 μs (4 V, 100 kHz input) were set for the 10 mM and 20 mM AsymPolPOK samples. A bandwidth of 80 MHz and chirp period of 10 μs (2 V, 100 kHz input), and bandwidth of 114 MHz and chirp period of 20 μs (1 V, 50 kHz input) were used for the 10 mM and 20 mM AMUPol samples, respectively.

Microwave power dependence of chirped and CW DNP

The microwave power reaching the sample can be attenuated using a quasi optical system including a Martin-Puplett interferometer[4] which is installed between the gyrotron and the DNP probe. This is achieved by adjusting a rotating wire grid polarizer.

The dependence of the microwave power on the chirped and CW DNP was analyzed for the 20 mM AMUPol sample at MAS frequencies of 7.7 kHz and 400 Hz (Fig. S7 a) and b)). At 7.7 kHz, saturation of the DNP enhancement was observed at around 10 W incident at the sample while saturation was achieved at 3 W for the slower spinning of 400 Hz. Furthermore,

chirps had no effect on the enhancement at microwave powers below saturation, (<4 W) at 7.7 kHz MAS. The effect of the microwave power on chirped and CW DNP was analysed at similar MAS frequencies for the 10 mM AsymPolPOK sample (Fig. S7 c) and d)). At 8 kHz MAS there is no significant difference observed between chirped and CW DNP at the lower power levels (<7 W) while at the lower MAS frequencies the microwave chirps lead to improved signal intensities even at 1.6 W. In general, higher power results in a bigger difference between the chirped and CW DNP signals.

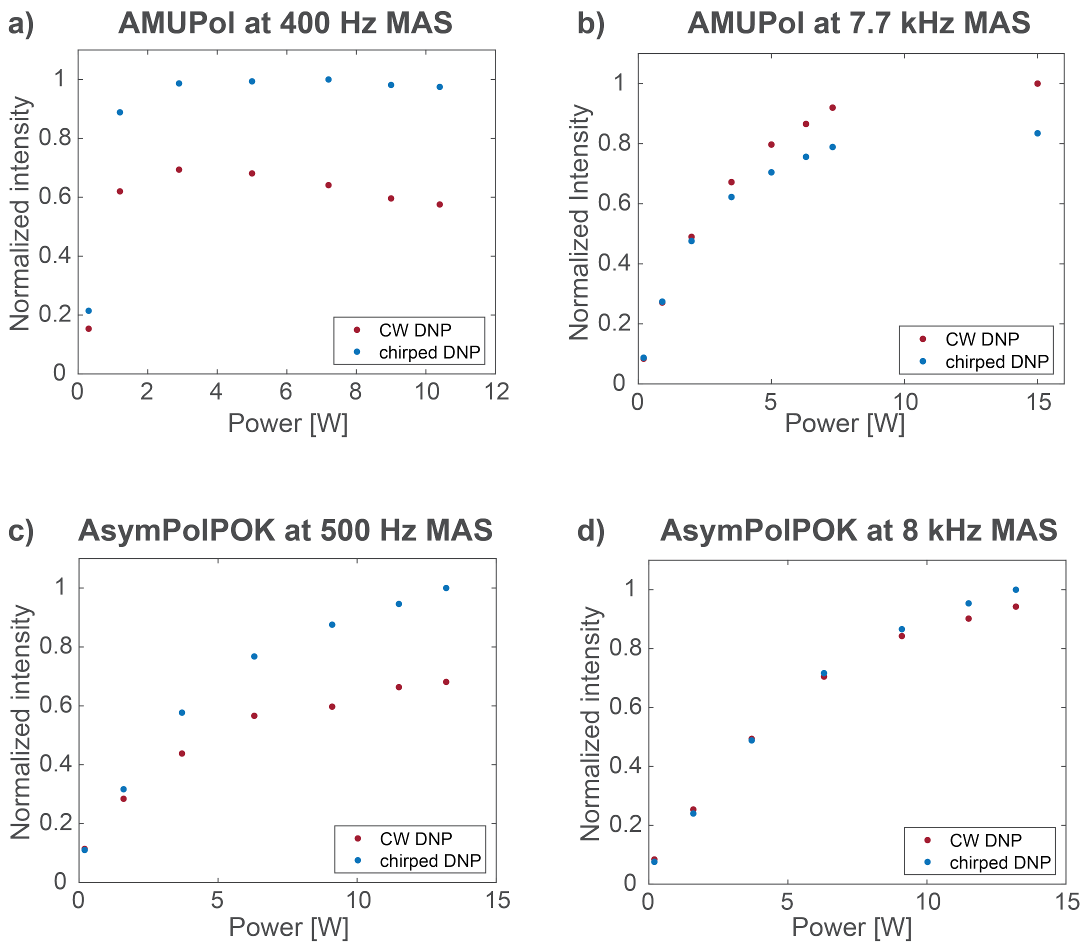

Fig. S7: Normalized ^{1}H signal intensities of the 20 mM AMUPol sample (**a)** and **b)**) and 10 mM AsymPolPOK sample (**b)** and **d)**) using CW and chirped DNP as a function of the power at the sample. Experiments were performed at different rotor spinning frequency of **a)** 0.4 kHz, **b)** 7.7 kHz, **c)** 0.5 kHz, and **d)** 8 kHz. The chirps correspond to 50 kHz, 1V and 100 kHz, 4V sinusoidal frequency modulation for AMUPol and AsymPolPOK, respectively.

Sweep parameter optimization

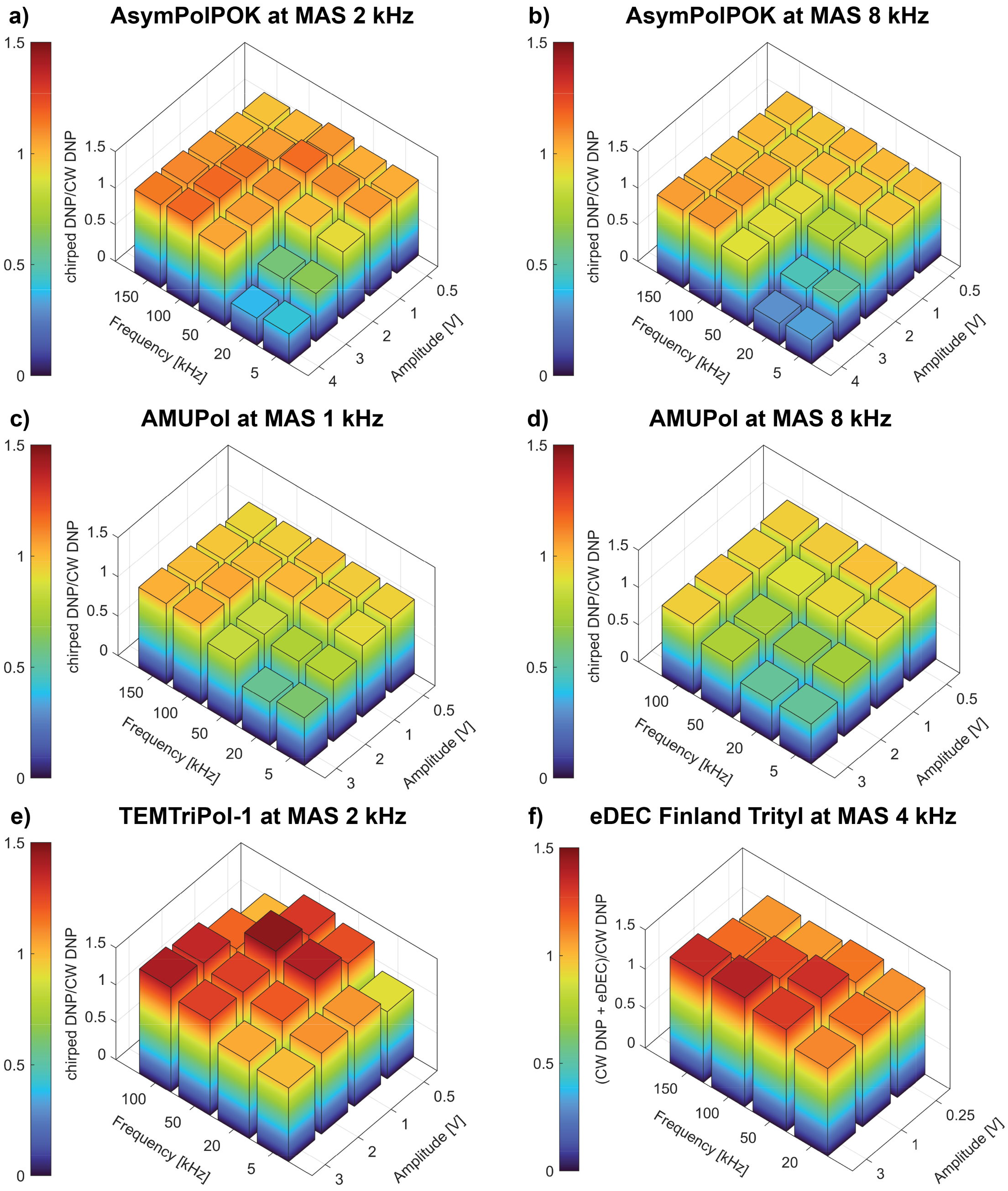

Fig. S8: Optimization of DNP chirp parameters and electron decoupling (eDEC) parameters. ^{1}H signal intensities of the frequency-chirped DNP experiments normalized with respect to CW DNP using different sweep parameters (frequency and amplitude) for **a)** 10 mM AsymPolPOK sample at 2 kHz MAS frequency, **b)** 10 mM AsymPolPOK sample spinning at 8 kHz, **c)** 10 mM AMUPol sample spinning at 1 kHz, **d)** 10 mM AMUPol sample spinning at 8 kHz, and **e)** 12 mM TEMTriPol-1 sample at 2 kHz MAS frequency. Values over 1 denote improvement. **f)** ^{13}C signal intensities of the Finland trityl sample spinning at 4 kHz, with sinusoidal electron decoupling. The spectra are normalized with respect to CW DNP experiments without electron decoupling, which illustrates that electron decoupling results in signal improvement for all sets of parameters.

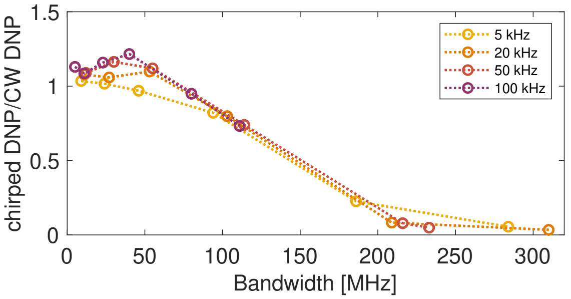

Fig. S9: Normalized ^{13}C signal intensities of the frequency-chirped DNP experiments compared to CW DNP using different sweep parameters for the Finland trityl sample at 4 kHz MAS frequency as a function of the microwave bandwidth. (same data as in Fig. 3 **c)**)

DNP profile of Finland Trityl

The line shape analysis of DNP profiles can reveal underlying DNP mechanisms. Fig. S10 shows the DNP profiles of 10 mM and 40 mM Finland trityl with 4 M ^{13}C,^{15}N-labelled urea in DNP juice. The negative and positive lobes of the DNP profile being closer to each other for the 40 mM concentration compared to the 10 mM concentration suggests that at 40 mM the solid effect and cross effect are both present.[5,6]

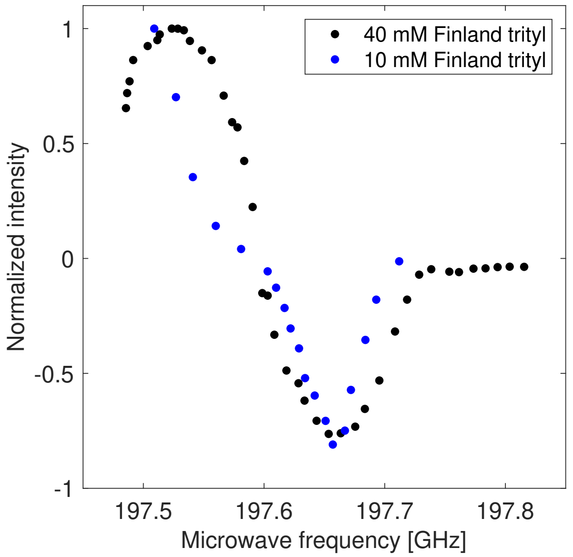

Fig. S10: ^{13}C DNP enhancement profile of 40 mM and 10 mM Finland trityl, 4 M ^{13}C,^{15}N-labelled urea in DNP juice, spinning at 4 kHz.

References

(1) Scott, F. J.; Alaniva, N.; Golota, N. C.; Sesti, E. L.; Saliba, E. P.; Price, L. E.; Albert, B. J.; Chen, P.; O'Connor, R. D.; Barnes, A. B. A versatile custom cryostat for dynamic nuclear polarization supports multiple cryogenic magic angle spinning transmission line probes. *J. Magn. Reson.* **2018**, *297*, 23–32.

(2) Mentink-Vigier, F.; Dubroca, T.; Van Tol, J.; Sigurdsson, S. T. The distance between g-tensors of nitroxide biradicals governs MAS-DNP performance: The case of the bTurea family. *J. Magn. Reson.* **2021**, *329*.

(3) Mentink-Vigier, F.; Mathies, G.; Liu, Y.; Barra, A. L.; Caporini, M. A.; Lee, D.; Hediger, S.; Griffin, R.; De Paëpe, G. Efficient cross-effect dynamic nuclear polarization without depolarization in high-resolution MAS NMR. *Chem. Sci.* **2017**, *8*, 8150–8163.

(4) Millen, M.; Pagonakis, I. G.; Björgvinsdóttir, S.; Alaniva, N.; Barnes, A. B. Control and Manipulation of Microwave Polarization and Power of a Frequency-Agile 198 GHz Gyrotron for Magnetic Resonance. *J. Infrared Millim. Terahertz Waves* **2023**, *44*, 281–296.

(5) Shimon, D.; Hovav, Y.; Feintuch, A.; Goldfarb, D.; Vega, S. Dynamic nuclear polarization in the solid state: A transition between the cross effect and the solid effect. *Phys. Chem. Chem. Phys.* **2012**, *14*, 5729–5743.

(6) Banerjee, D.; Shimon, D.; Feintuch, A.; Vega, S.; Goldfarb, D. The interplay between the solid effect and the cross effect mechanisms in solid state 13C DNP at 95 GHz using trityl radicals. *J. Magn. Reson.* **2013**, *230*, 212–219.
